# Correction: Schlotheuber, A.; Hosseinpoor, A.R. Summary Measures of Health Inequality: A Review of Existing Measures and Their Application. *Int. J. Environ. Res. Public Health* 2022, *19*, 3697

**DOI:** 10.3390/ijerph19126969

**Published:** 2022-06-07

**Authors:** Anne Schlotheuber, Ahmad Reza Hosseinpoor

**Affiliations:** Department of Data and Analytics, World Health Organization, 1211 Geneva, Switzerland; schlotheuberan@who.int

Several errors were introduced after proofreading, and the authors hence wish to make the following corrections to this paper [1]:

## Figure Legend

In the original publication, there was a mistake in the legend for Figure 1. The figure note “* Weighted measure. ** Weighted or unweighted measure.” was redundant as this information is included in Figure 1. The corrected legend is shown below.

Figure 1. Overview of summary measures of health inequality.

## Error in Tables

In the original publication, there was a mistake in Tables A7 and A9. Several numbers in the tables were missing a decimal. The corrected Tables A7 and A9 are shown below. 

**Table A7:** Economic-related inequality in three reproductive, maternal and child health indicators: Indonesia (DHS: 1997, 2007 and 2017).

Health Indicator	Summary Measure	1997	2007	2017
Births attended by skilled health personnel	Difference (D)	67.4 (63.3–71.6)	49.5 (45.7–53.3)	23.6 (20.5–26.8)
	Ratio (R)	4.1 (3.6–4.7)	2.1 (1.9–2.2)	1.3 (1.3–1.4)
	Absolute concentration index (ACI)	12.9 (12.1–13.7)	9.8 (9.1–10.5)	4.3 (3.7–4.9)
	Relative concentration index (RCI)	25.3 (24.4–26.1)	13.1 (12.8–13.3)	4.7 (4.6–4.7)
	Slope index of inequality (SII)	72.9 (70.8–74.9)	59.9 (57.5–62.3)	31.6 (28.9–34.3)
	Relative index of inequality (RII)	6.1 (5.6–6.7)	2.6 (2.5–2.8)	1.5 (1.4–1.5)
	Population attributable risk (PAR)	38.3 (36.7–39.9)	21.2 (19.3–23.0)	7.6 (6.1–9.2)
	Population attributable fraction (PAF)	75.1 (72.0–78.3)	28.3 (25.8–30.7)	8.3 (6.6–10.0)
Demand for family planning satisfied-use of modern and traditional methods	Difference (D)	10.3 (7.3–13.2)	8.0 (5.4–10.6)	–0.4 (–2.2–1.4)
	Ratio (R)	1.1 (1.1–1.2)	1.1 (1.1–1.1)	1.0 (1.0–1.0)
	Absolute concentration index (ACI)	1.8 (1.3–2.3)	1.3 (0.9–1.7)	–0.2 (–0.5–0.2)
	Relative concentration index (RCI)	2.1 (2.1–2.1)	1.5 (1.5–1.5)	–0.2 (–0.2–0.2)
	Slope index of inequality (SII)	11.4 (9.6–13.2)	8.1 (6.5–9.7)	–1.0 (–2.4–0.5)
	Relative index of inequality (RII)	1.1 (1.1–1.2)	1.1 (1.1–1.1)	1.0 (1.0–1.0)
	Population attributable risk (PAR)	4.1 (2.9–5.4)	1.5 (0.3–2.6)	0.0 (–0.9–0.9)
	Population attributable fraction (PAF)	4.8 (3.4–6.2)	1.7 (0.4–2.9)	0.0 (–1.1–1.1)
Measles immunisation coverage among one-year-olds	Difference (D)	26.1 (19.3–32.9)	21.7 (14.9–28.5)	12.0 (6.5–17.5)
	Ratio (R)	1.4 (1.3–1.6)	1.3 (1.2–1.5)	1.2 (1.1–1.3)
	Absolute concentration index (ACI)	4.8 (3.5–6.1)	4.1 (2.8–5.3)	2.5 (1.5–3.5)
	Relative concentration index (RCI)	6.8 (6.5–7.0)	5.3 (5.1–5.5)	3.2 (3.1–3.2)
	Slope index of inequality (SII)	29.9 (24.5–35.2)	25.1 (19.9–30.2)	15.5 (10.7–20.4)
	Relative index of inequality (RII)	1.5 (1.4–1.7)	1.4 (1.3–1.5)	1.2 (1.1–1.3)
	Population attributable risk (PAR)	14.3 (11.0–17.6)	8.5 (5.2–11.8)	4.9 (1.9–7.8)
	Population attributable fraction (PAF)	20.2 (15.5–24.8)	11.1 (6.8–15.4)	6.2 (2.4–9.9)

Data source: Summary measures calculated using the WHO Health Equity Assessment Toolkit (HEAT), based on disaggregated data from the WHO Health Equity Monitor database (2021 update).

**Table A9:** Subnational regional inequality in three reproductive, maternal and child health indicators: Indonesia (DHS 2017).

Summary Measure	Births Attended by Skilled Health Personnel	Demand for Family Planning Satisfied-Use of Modern and Traditional Methods	Measles Immunisation Coverage among One-Year-Olds
Difference (D)	35.8 (22.0–49.6)	29.4 (20.2–38.7)	40.0 (29.1–50.8)
Ratio (R)	1.6 (1.3–1.9)	1.5 (1.3–1.7)	1.7 (1.5–2.1)
Between-group variance (BGV)	50.4 (31.7–69.2)	18.1 (13.4–22.9)	83.9 (49.9–117.8)
Between-group standard deviation (BGSD)	7.1 (–11.6–25.8)	4.3 (–0.5–9.0)	9.2 (–24.8–43.1)
Coefficient of variation (COV)	7.8 (–11.0–26.5)	5.0 (0.2–9.7)	11.6 (–22.3–45.6)
Mean difference from mean (unweighted) (MDMU)	6.6 (6.1–7.7)	5.4 (5.2–6.2)	7.9 (7.3–10.1)
Mean difference from mean (weighted) (MDMW)	5.3 (4.8–6.5)	2.9 (2.7–3.6)	7.6 (6.4–9.7)
Mean difference from best group (unweighted) (MDBU)	8.4 (7.0–9.4)	6.8 (6.3–7.5)	14.7 (13.4–16.4)
Mean difference from best group (weighted) (MDBW)	10.3 (9.2–11.3)	9.2 (8.7–10.0)	14.3 (13.3–16.4)
Index of disparity (unweighted) (IDISU)	7.2 (6.6–8.6)	6.3 (6.0–7.3)	10.1 (9.2–13.0)
Index of disparity (weighted) (IDISW)	5.8 (5.2–7.1)	3.4 (3.2–4.2)	9.6 (8.1–12.3)
Theil index (TI)	3.1 (3.1–3.1)	1.3 (1.3–1.3)	7.0 (7.0–7.0)
Mean log deviation (MLD)	3.3 (3.3–3.3)	1.3 (1.3–1.3)	7.4 (7.4–7.4)
Population attributable risk (PAR)	8.4 (1.5–15.3)	6.8 (–5.4–18.9)	14.7 (3.6–25.8)
Population attributable fraction (PAF)	9.2 (1.6–16.7)	7.9 (–6.2–22.1)	18.6 (4.6–32.7)

Data source: Summary measures calculated using the WHO Health Equity Assessment Toolkit (HEAT), based on disaggregated data from the WHO Health Equity Monitor database (2021 update).

## Text Correction

There was an error in the original publication. There were several formatting errors in Section 3. Summary Measures of Health Inequality, including sub-section headers should have been italic; summary measure names (headers) should have been bold; headers for “Definition”, “Calculation” and “Interpretation” should not have been numbered; text for “Definition”, “Calculation” and “Interpretation” should not have been italic. A correction has been made to 3. Summary Measures of Health Inequality. Please refer to the publication for the corrected text.

The authors apologize for any inconvenience caused and wish to state that the scientific conclusions are unaffected. The original publication has also been updated.
